# Targeting Overtreatment of Asymptomatic Bacteriuria in the Emergency Department: Results from a Quasi-Experimental Clinical Pharmacist-Led Program Based on Education and Audit

**DOI:** 10.3390/antibiotics14121261

**Published:** 2025-12-14

**Authors:** Alvaro Monje, Laura Escolà-Vergé, Alba Rivera, Sergio Herrera, Adrián Plaza, Pol Duch-Llorach, Virginia Pomar, Nerea Roch, Laia Rouras, Joaquín López-Contreras, Jesús Ruiz Ramos

**Affiliations:** 1 Pharmacy Department, Hospital de la Santa Creu i Sant Pau, Carrer de Sant Quintí, 89, 08025 Barcelona, Spain; alvaro.monjelopez96@gmail.com (A.M.);; 2 Departament de Medicina, Universitat Autònoma de Barcelona, Unitat Docent de la Vall d’Hebron Mòdul Sud. Pavelló Docent, Passeig de la Vall d’Hebron, 119-129, 08035 Barcelona, Spain; 3 Infectious Diseases Department, Hospital de la Santa Creu i Sant Pau, Carrer de Sant Quintí, 89, 08025 Barcelona, Spain; 4 Institut de Recerca Sant Pau (IR SANT PAU), Sant Quintí 77, Horta-Guinardó, 08041 Barcelona, Spain; 5 Centro de Investigación Biomédica en Red de Enfermedades Infecciosas (CIBERINFEC), Instituto de Salud Carlos III (ISCIII), Av. Monforte de Lemos, 3-5. Pabellón 11, Planta 0, 28029 Madrid, Spain; 6 Microbiology Department, Hospital de la Santa Creu i Sant Pau, Carrer de Sant Quintí, 89, 08025 Barcelona, Spain; 7 Genetics and Microbiology Department, Universitat Autònoma de Barcelona, 08025 Barcelona, Spain; 8 Emergency Department, Hospital de la Santa Creu i Sant Pau, Carrer de Sant Quintí, 89, 08025 Barcelona, Spain

**Keywords:** asymptomatic bacteriuria, emergency department, audit and feedback, urinary tract infection, urine culture

## Abstract

**Background:** Asymptomatic bacteriuria (ASB) is frequently overtreated in emergency departments (EDs), contributing to antimicrobial resistance without improving clinical outcomes. The rapid pace of clinical decision-making and high patient turnover in the ED further predispose clinicians to unnecessary antibiotic prescribing. **Methods:** A quasi-experimental study was conducted in the ED of a tertiary hospital in Barcelona, Spain, from January 2024 to September 2025. The intervention included targeted education for ED staff and daily audit-feedback on antibiotic prescriptions for suspected ASB. The outcomes were the following variables, compared between study periods: cases of ASB with unnecessary antibiotic treatment per month, antimicrobial consumption, urine culture (UC) requests, 30-day return visits to the ED for urinary tract infection, and 30-day all-cause mortality for safety assessment. **Results:** A total of 93 patients with suspected ASB in the pre-intervention period and 102 patients in the intervention period were included. The median cases of ASB with unnecessary antibiotic treatment per month decrease from 19 (IQR 16–26) in the pre-intervention period to 9 (IQR 9–13) in the intervention period (*p* = 0.018). Antimicrobial consumption declined: meropenem and imipenem decreased from 5.5 to 3.0 DDD/1000 admissions, ertapenem from 5.6 to 3.1, and ceftriaxone from 35.0 to 24.1. UC requests fell by 16.1%. Clinical safety outcomes did not differ significantly between periods: 30-day return visit to the ED for UTI with the same isolate dropped from 8.6% to 1.9% (*p* = 0.076), overall UTI return visits to the ED dropped from 11.8% to 5.9% (*p* = 0.225), and 30-day mortality remained stable (8.6% vs. 4.9%, *p* = 0.455). **Conclusions:** These findings support the use of combined educational and audit–feedback strategies as effective and safe Antimicrobial Stewardship interventions in high-intensity clinical environments such as the ED, as they reduce inappropriate antibiotic use and unnecessary UC requests without compromising patient safety.

## 1. Introduction

Asymptomatic bacteriuria (ASB) is frequently associated with inappropriate antibiotic prescribing, contributing to the emergence of antimicrobial resistance [1,2]. According to the 2019 Infectious Diseases Society of America guidelines [3], antibiotic treatment is only indicated for pregnant women and patients undergoing urological procedures. Conversely, in institutionalized patients, routine treatment of ASB has shown no clinical benefit and is associated with an increased risk of adverse events [4].

Emergency departments (EDs) are characterized by high patient volumes and substantial time pressure, conditions that challenge accurate diagnostic assessment and antimicrobial decision-making. Many patients are discharged with empiric outpatient antibiotic prescriptions before culture and susceptibility results become available [5]. This challenge is compounded by high turnover among resident and attending physicians, which may reduce consistency in diagnostic and therapeutic approaches [6].

Approximately 27% to 45% of urine positive culture (UC) results correspond to ASB, depending on the clinical setting [1,7]. In patients presenting to ED, clinical history-taking is often complex due to cognitive impairment and altered mental status at presentation [3]. These factors may increase the number of microbiological diagnostic tests requested and complicate their clinical interpretation, leading to unnecessary antibiotic prescriptions and contributing to antimicrobial resistance increase in this population.

The primary objective of this study was to evaluate the impact of a pharmacist-led educational and audit–feedback program on reducing overtreatment of ASB in the ED.

## 2. Results

**Figure 1:**
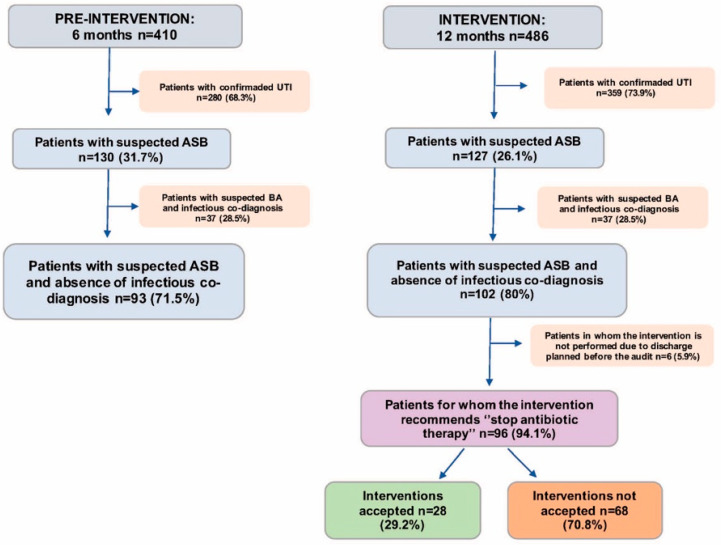
Flowchart of patients evaluated and included in the study. ASB: Asymptomatic bacteriuria; UTI: Urinary Tract Infection.

The total number of patients evaluated and included in both periods is represented in Figure 1 (number of patients with antibiotics and confirmed urinary tract infection [UTI], patients with antibiotics and suspected ASB, patients with antibiotics and suspected ASB without an infectious co-diagnosis, the number of “stop antibiotic therapy” interventions in the intervention group, as well as the acceptance rate).

### 2.1. Data Collection

During the pre-intervention period, a total of 410 patients were identified: 280 (75.1%) were excluded (they met UTI criteria) and 130 (31.7%) patients were classified as potential ASB, of whom 93 (71.5%) patients had no associated infectious co-diagnosis.

In the intervention period, 486 patients were evaluated, with a total of 359 (73.9%) patients with UTI criteria and 127 (26.1%) patients with suspected ASB, of whom 102 (80%) patients had no simultaneous infectious diagnosis.

In 96 patients (94.1%), discontinuation of antibiotic therapy was recommended to the ED physician and documented in the patient’s medical record. However, in 6 patients (5.9%), no recommendation was made because the patient was discharged with antibiotic prescription before audit and feed-back. The recommendation was accepted in 28/96 cases (29.2%). The main reason for rejection was that the outpatient treatment plan had already been established and communicated to the patient, their family or receiving care teams (13/28, 46%).

### 2.2. Description of the Sample of Patients with ASB

A total of 195 patients receiving active antibiotic therapy with suspected ASB and without any concurrent infectious diagnosis justifying antibiotic treatment were included (93 in the pre-intervention group and 102 in the intervention group). The demographic and clinical characteristics of both groups are summarized in Table 1. The mean age was similar between groups. A higher proportion of males was observed in the pre-intervention group (25.4% vs. 7.1%, *p* < 0.001). A Charlson index > 6 was also more frequent in the pre-intervention group (16.2% vs. 7.1%, *p* = 0.02), as well as a higher prevalence of heart failure (24.6% vs. 9.5%, *p* < 0.001). Clinically, there was a greater proportion of confusional syndrome in the pre-intervention group (92.3% vs. 23.7%, *p* < 0.001).

### 2.3. Antibiotic Consumption

Table 2 summarizes total antibiotic consumption and the use of total, non-ertapenem carbapenems (imipenem and meropenem), ertapenem, and ceftriaxone expressed as DDD/1000 admissions by quarter. Total antibiotic consumption decreased from 53.7 DDD/1000 admissions in January–March 2024 to 46.9 in January–March 2025 (−12.6%) and from 51.8 in April–June 2024 to 40.9 in April–June 2025 (−21.0%). A similar reduction was observed in the July–September quarters between 2024 and 2025, with a decline from 53.4 to 41.4 DDD/1000 (−22.5%). In the October–December comparison in 2023 and 2024, total consumption decreased from 56.5 in 2023 to 45.7 in 2024 (−19.1%).

In subgroup analysis, non-ertapenem carbapenems showed a sustained decline across the study period, falling from 5.5 to 3.0 DDD/1000 admissions between Jul–Sep 2023 and Apr–Jun 2025, with a modest increase in the final quarter (3.9 DDD/1000). Ertapenem displayed a similar pattern, peaking in Jul–Sep 2024 (5.6 DDD/1000) and subsequently decreasing to 3.1 DDD/1000 from Oct–Dec 2024 onward. Finally, Ceftriaxone consumption demonstrated the largest absolute reduction, declining from 35.0 to 24.1 DDD/1000 over the same interval and remaining stable in the post-intervention period (24.4 DDD/1000).

**Figure 2:**
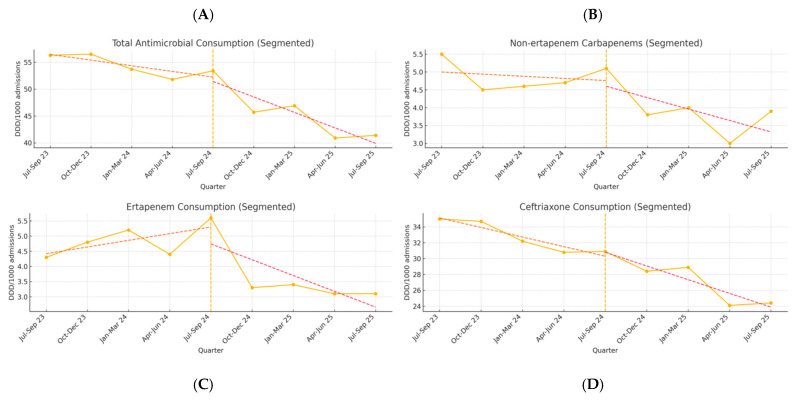
Interrupted time series of antimicrobial consumption in the Emergency Department (January 2023–September 2025). Quarterly antimicrobial consumption expressed as DDD/1000 admissions for: (**A**) total antibiotics, (**B**) non-ertapenem carbapenems (meropenem and imipenem), (**C**) ertapenem, and (**D**) ceftriaxone. The vertical dashed line marks the beginning of the intervention period (July 2024). Data points represent observed quarterly values.

Using an Interrupted Time Series Analysis, the results of the segmented regression analysis for all antimicrobial groups are presented in Table 3 and Figure 2. Following the intervention, there was a non-significant immediate level change (β_2_ = +5.82, *p* = 0.587), indicating no abrupt step change in consumption at the intervention point. However, the trend change coefficient (β_3_ = −2.73, *p* = 0.462) suggested an acceleration in the rate of decline post-intervention. The combination of the baseline trend and the post-intervention trend change (β_1_ + β_3_ = −5.26 DDD/100 bed-days per quarter) indicates a steeper decline in the post-intervention period. When examining specific antimicrobial groups, several notable findings emerged. For ertapenem, a statistically significant change in the post-intervention trend was observed (β_3_ = −0.71, *p* = 0.043), indicating that the intervention was associated with an accelerated decline in ertapenem consumption. Similarly, non-ertapenem carbapenems overall showed a near-significant trend change (β_3_ = −0.85, *p* = 0.075), suggesting a clinically meaningful effect that approached statistical significance. Ceftriaxone showed a significant baseline declining trend (β_1_ = −1.52, *p* = 0.005).

### 2.4. Number of Urine Cultures Requests and Urine Cultures

During the intervention period, a reduction in UC requests was observed. The mean number of UC processed decreased by 16.1%, from 1063 samples per month in the pre-intervention period to 892 samples per month after implementation of the Antimicrobial Stewardship (AMS) intervention. This decline was accompanied by a reduction in negative cultures (−17.3% per month), positive cultures (−12.2%), and total microbiological isolates (−16.5%). Isolates considered non-uropathogenic flora (including *Candida* spp., Gram-positive cocci, and commensal organisms) showed the largest relative reduction (−20.8%), while uropathogenic isolates decreased by 15.9% per month, consistent with the overall decrease in culture positivity. When comparing both groups, a decrease in the total number of UC requests per month was observed.

**Figure 3:**
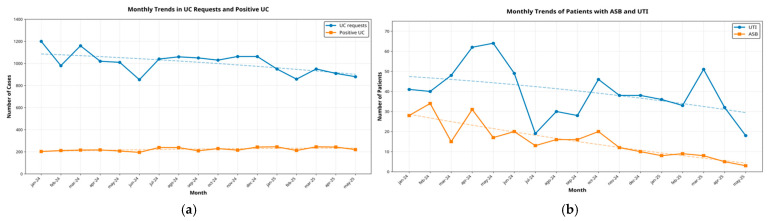
(**a**) Monthly evolution of the number of Urine Culture (UC) requests in blue line and positive Urine Cultures in orange line. (**b**) Patients with Urinary Tract Infection (UTI) in blue line and patients with Asymptomatic Bacteriuria (ASB) in orange line.

Figure 3 shows the monthly evolution of the number of UC requests and positive UC (Figure 3a), as well as patients with ASB and UTI (Figure 3b) between January 2024 and June 2025. In Figure 3a, UC requests decreased from approximately 1100–1200 per month to around 900–950, while positive UC per month remained stable over time. In Figure 3b, the number of UTI diagnoses shows variability over time, with a gradual downward trend from approximately 45–50 cases per month at baseline to around 20–30 cases in the final months. ASB diagnoses also decreased over the study period, from approximately 20–25 to around 5–10 cases per month. Both series show an overall similar downward trend.

### 2.5. Clinical Outcomes

Clinical outcomes are presented in Table 4. The average monthly number of ASB cases who received unnecessary antibiotic therapy decreased from a median of 19 patients/month (IQR 16–26) in the pre-intervention period toa median of 9 patients/month (IQR 9–13) during the intervention period (*p* = 0.018). Trends are shown in Figure 3b.

During the pre-intervention phase, 11/93 patients (11.8%) who had been discharged with antibiotics returned to the ED for UTI, of whom 8/93 (8.6%) had the same microbiological isolate. Eight patients (8.6%) died within 30 days. Only two of these deaths occurred in patients who had reconsulted for UTI with the same isolate, and none of the deaths were attributable to infection. During the intervention phase, 6 patients (5.9%) with ASB returned to the ED within 30 days, and 2 (2%) had a UTI caused by the same isolate as the ASB. Of these 6 patients, all had belonged to the group in which the recommendation to discontinue antibiotic therapy had been rejected and antibiotics were continued, while no 30-day UTI revisits occurred among patients in whom antibiotics were stopped following the intervention. Five patients (4.9%) died within 30 days, none due to infection.

No statistically significant differences were observed between groups in 30-day UTI return visits with the same isolate (8.6% vs. 2.0%, *p* = 0.076), overall UTI return visits (11.8% vs. 5.9%, *p* = 0.225), or 30-day mortality (8.6% vs. 4.9%, *p* = 0.455).

## 3. Discussion

While previous pharmacist-led programs focused solely on educational approaches to reduce antibiotic prescribing for ASB [8,9], our study evaluated a combined strategy involving education and daily audit-feedback in the ED. Our intervention showed a decreased antimicrobial consumption, and a decline in the number of unnecessary UC requests, without compromising clinical outcomes such as ED revisits or mortality. The multifaceted design combining education, audit and feedback conforms to current AMS frameworks, which emphasize iterative reinforcement, diagnostic refinement, and clinician behavioral change as key drivers of sustained improvement [10].

Regarding the study population, both groups had comparable demographic profiles, consisting predominantly of older, multimorbid, and female patients, similar to populations described in previous studies [11]. However, there were fewer patients with confusional syndrome in the intervention group. This difference likely reflects a change in the diagnostic approach of ED physicians. The educational intervention focused on the definition of ASB emphasizing that delirium alone should not prompt UC in the absence of urinary symptoms [3] and this idea was reinforced during the feedback process. Therefore, during the intervention, clinicians were probably less prone to routinely request UC in patients presenting with isolated confusional syndrome in the absence of urinary symptoms or systemic signs of infection, and increasingly prioritized the investigation of non-infectious causes of delirium [12].

Regarding the audit–feedback component, the relatively low acceptance rate of deprescribing recommendations was notable. The barriers to accepting such recommendations may vary and include diagnostic uncertainty, perceived clinical risk, limited post-discharge follow-up, and the high-pressure ED environment, where clinicians tend to prioritize immediate safety over deprescribing [13,14,15]. In our cohort, patients had already been discharged or had plans for discharge or transfer to continue parenteral antimicrobial treatment, making reversal of therapeutic decisions structurally complex.

Nonetheless, antimicrobial consumption declined despite the modest acceptance of direct recommendations, suggesting broader behavioral effects of the intervention. Notably, the intervention appeared to have the most significant impact on ertapenem consumption. This finding is clinically relevant, as our institution shows a high prevalence of ESBL-producing Enterobacterales in UC (approximately 25–30%), a context in which ertapenem is frequently used as empirical or targeted therapy for UTI. This pattern suggests that the intervention influenced subsequent prescribing behavior even when individual deprescribing recommendations were not adopted.

There have been other studies on ASB that have shown great reductions in unnecessary antibiotic treatment in tertiary-care academic hospital settings [16,17]. For example, Irfan et al. [16]. implemented a monthly educational intervention for medical residents to reduce inadequate ASB treatment with inappropriate treatment rates declining from 58.8% to 8%. On the other hand, other laboratory-based diagnostic stewardship intervention [17] demonstrated that a laboratory-based diagnostic stewardship intervention consisting of no longer routinely reporting UC results from non-catheterized medical and surgical inpatients in a tertiary teaching hospital reduced inappropriate treatment from 48% to 12% (*p* = 0.002). Both strategies focused on reducing antimicrobial prescribing, rather than withdrawing antibiotics already initiated, which appears to be more challenging according to our cohort.

In fact, most audit-and-feedback interventions do not provide immediate feedback but instead deliver periodic reports on the adequacy of individual prescriptions, often including comparisons with other prescribers [18,19]. However, acceptance rates for individual antimicrobial withdrawal recommendations, particularly in the ED, have not been extensively explored [14,15]. Our findings highlight that real-time deprescribing recommendations in ED may face additional barriers compared with periodic feedback models in other clinical settings.

On the other hand, the intervention also enhanced diagnostic stewardship. Reductions in UC requests, along with fewer negative or non-uropathogen cultures, suggest more judicious diagnostic practices, particularly in patients presenting with confusional syndrome. Importantly, the monthly number of ASB patients receiving unnecessary antibiotic therapy declined significantly during the intervention period, highlighting the effectiveness of integrating diagnostic and AMS strategies. These findings align with a recent multicenter study using educational strategies among 14,572 hospitalized patients found that, over 3 years, the percentage of patients with a positive UC who had ASB (diagnostic stewardship metric) declined from 34.1% to 22.5%, whereas the percentage of patients with ASB who received antibiotics remained relatively stable, from 82.0% to 76.3% [20]. This suggests that probably the most effective strategy lies in improving diagnostic stewardship (reducing unnecessary UC) rather than withholding already initiated antibiotic prescriptions for patients with ASB [21].

From a clinical perspective, the intervention was safe, with no evidence of harm associated with deprescribing. These findings are consistent with previous studies [22,23], which show that withholding antibiotics in cases of true ASB does not increase the risk of recurrent UTI or mortality. They also support the notion that treating ASB provides no clinical benefit and may, in fact, increase the risk of adverse events and contribute to antimicrobial resistance [24].

Regarding the sustainability of the intervention, a post-intervention phase without active audit or feedback was specifically included to explore whether prescribing changes would persist after withdrawal of the proactive strategy. Although a partial rebound in antibiotic consumption was observed, overall prescribing levels remained lower than during the pre-intervention period, suggesting a residual effect of the program. This finding is consistent with the concept that repeated educational and audit–feedback interventions may lead to sustained behavioral change through reinforcement of clinical reasoning and normalization of deprescribing practices [19,20]. Nevertheless, long-term sustainability beyond the three-month post-intervention period could not be assessed, and future studies and follow-up in the future should evaluate the durability of these effects over extended follow-up periods.

## 4. Materials and Methods

We conducted a quasi-experimental, before–after study with prospective data collection in the ED of a tertiary-care hospital with 650 beds in Barcelona, Spain, serving approximately 140,000 ED visits per year. It included a six-month pre-intervention observational and prospective period (January–June 2024), and a 12-month intervention and prospective period (July 2024–June 2025). All patients evaluated in the ED with a presumptive diagnosis of UTI and active antibiotic prescription were screened. Patient identification was performed through daily review of antibiotic prescriptions in the electronic prescribing system by clinical pharmacists, followed by a structured review of the electronic medical record. Patients meeting the diagnostic criteria for ASB, according to the IDSA guidelines [3], were included.

Patients were considered to have potential ASB if they had a positive UC (≥10^5^ CFU/mL) in the absence of UTI-related clinical criteria. UTI-related clinical criteria included the presence of local genitourinary symptoms (incontinence, dysuria, urgency, or frequency) and/or systemic signs suggestive of infection (fever, chills, hemodynamic instability, CRP > 50 mg/L and/or leukocytosis > 12,000 cells/μL) without another alternative explanation [25].

The intervention phase included an initial educational phase followed by a continuous audit–feedback phase (Table 5). The educational phase lasted one week and consisted of targeted sessions delivered to ED prescribers and nursing staff by the AMS team. For ED prescribers, a one-hour session was conducted on 23 May 2024, at 08:00. For nursing staff, multiple 30 min sessions were delivered between 21–23 May 2024, on different days and at different times to ensure coverage of all shifts (morning, afternoon, evening, and night) to maximize attendance. Content included updated ASB management guidelines, their application in the ED, criteria for UC ordering, how to obtain a urinary sample, and baseline results from the pre-intervention period. Attendance was recorded, with approximately 90% of nursing staff and 60% of physicians attending the educational sessions, representing around 80% of the regular ED workforce. All educational materials were subsequently distributed by institutional e-mail to the entire ED staff.

During the audit–feedback phase, conducted on weekdays over 12 months, clinical pharmacists performed a daily structured review and audit of all antibiotic prescriptions initiated during the previous 24 h for patients with suspected UTI. For cases with suspected ASB, feedback was provided directly to prescribers, and discontinuation of antibiotics was recommended when no exclusion criteria were identified (i.e., UTI criteria not reflected in the medical records or an alternative infectious focus). All interventions (whether accepted or rejected) as well as the clinical rationale for rejection were systematically documented. Data regarding acceptance or rejection by ED physicians were anonymized to preserve clinician confidentiality and avoid perceptions of punitive oversight by the AMS team.

To assess the sustainability of the intervention, a post-intervention phase without active audit or feedback was conducted during July–September 2025, in which antibiotic consumption was evaluated.

The primary outcomes were monthly number of ASB patients receiving unnecessary antibiotics, UC requests and antibiotic consumption in both periods. Given the safety concerns commonly associated with withholding antibiotic therapy in ED, secondary outcomes included 30-day ED return visits for UTI and 30-day all-cause mortality. 

Continuous variables were tested for normality using the Shapiro–Wilk test. As the monthly number of ASB cases with unnecessary antibiotic treatment showed a non-normal distribution, results are presented as median and interquartile range (IQR) and comparisons between pre-intervention and intervention periods were performed using the Mann–Whitney U test. Categorical variables (e.g., revisit rates, mortality, acceptance of recommendations) were compared using χ^2^ or Fisher’s exact test, as appropriate. A two-sided *p*-value < 0.05 was considered statistically significant. Statistical analyses were conducted using STATA 19.

Antimicrobial consumption was expressed as defined daily doses per 1000 ED admissions (DDD/1000 admissions). DDD/1000 admissions metric was selected as the main antimicrobial consumption indicator as it best reflects ED prescribing patterns for daily dosed antibiotics [26,27]. Antimicrobial consumption was analyzed by quarters, comparing the first (January–March 2024), second (April–June 2024), and third (July–September 2024) quarters with the corresponding periods in 2025. Due to the unavailability of 2025 data, antimicrobial consumption during the fourth quarter (October–December 2024) was compared with that of the fourth quarter of 2023.

An interrupted time series (ITS) analysis was conducted to evaluate the impact of a quality improvement intervention on antimicrobial consumption in the ED. We analyzed quarterly data on antimicrobial use from the first quarter of 2023 (January–March 23) to the second quarter of 2025 (April–June 25), providing a total of 10 data points spanning two and a half years. The institutional information system only allows extraction of antimicrobial consumption data at a quarterly level. The model was fitted using ordinary least squares regression. We performed a segmented regression analysis, which is the standard statistical approach for ITS studies, to estimate the changes in antimicrobial consumption following the intervention. Model fit was assessed using the coefficient of determination (R^2^). A *p*-value < 0.05 was considered statistically significant. To visually support the interrupted time series analysis, quarterly antimicrobial consumption data were displayed as time-series line plots (Figure 2), with the intervention point indicated by a vertical dashed line at Q3 2024. Separate plots were generated for total antimicrobial consumption, non-ertapenem carbapenems, ertapenem, and ceftriaxone.

## 5. Limitations

Despite the positive outcomes, several limitations should be acknowledged. First, as a single-center study with a relatively small sample size, the external validity may be limited, and the study may be underpowered to detect certain differences. Second, the long-term sustainability of the intervention beyond the short post-intervention follow-up period could not be assessed. Third, individual data regarding the prescribing practices of ED physicians were not collected, so the impact of the audit-and-feedback strategy on future prescriptions at the individual level remains unknown. Finally, as an observational study, causality cannot be definitively established. Additionally, potential variations in ED patient volume and case mix over time may have influenced the results and should be considered as residual sources of confounding. However, the temporal relationship between the intervention and the observed outcomes, together with consistency with previous AMS studies, supports the plausibility of an intervention effect.

## 6. Conclusions

Although the implementation of an education and audit–feedback program in the ED was associated with a reduction in antimicrobial consumption and unnecessary diagnostic testing without compromising short-term patient safety, our findings should be interpreted with caution given the study’s observational design, potential residual confounding, and relatively small sample size. While direct acceptance of deprescribing recommendations was limited, the combined effect of structured education and sustained audit–feedback was associated with progressive improvements in prescribing behavior over time. Collectively, these findings support the safety of antibiotic deprescribing in ASB and reinforce the value of educational interventions as a cornerstone strategy for optimizing antimicrobial use and mitigating resistance in ED settings. Larger multicenter studies with more robust analytical approaches are needed to confirm these findings and to better quantify their impact while accounting for potential confounding factors.

## Figures and Tables

**Table 1:** Demographic and clinical characteristics of patients in both periods.

Variables	Pre-Intervention Group (n = 93)	Intervention Group (n = 102)	*p*-Value
Demographic characteristics			
Mean age (years ± SD)	84.3 ± 9.2	84.0 ± 11.4	0.841
Male sex (%)	33 (25.4%)	10 (7.1%)	**<0.001**
Charlson index > 3 (%)	35 (26.9%)	39 (30.7%)	0.492
Charlson index > 6 (%)	21 (16.2%)	9 (7.1%)	**0.021**
Comorbidities			
Cognitive impairment	92 (70.8%)	85 (66.9%)	0.521
Chronic kidney disease	27 (20.8%)	32 (25.2%)	0.413
Chronic Obstructive Pulmonary Disease	9 (6.9%)	7 (5.5%)	0.663
Diabetes mellitus	44 (33.8%)	38 (29.9%)	0.502
Heart failure	32 (24.6%)	12 (9.4%)	**<0.001**
Institutionalized in nursing home	60 (46.2%)	48 (37.8%)	0.181
Confusional syndrome	120 (92.3%)	30 (23.7%)	**<0.001**

Note: Bold *p*-values indicate statistical significance (*p* < 0.05).

**Table 2:** Antimicrobial consumption expressed in Defined Daily Dose (DDD)/1000 admissions.

DDD/1000 Admissions	Jul–Sep 2023	Oct–Dec 2023	Jan–Mar 2024	Apr–Jun 2024	Jul–Sep 2024	Oct–Dec 2024	Jan–Mar 2025	Apr–Jun 2025	Jul–Sep 2025
Total	56.3	56.5	53.7	51.8	53.4	45.7	46.9	40.9	41.4
Non-ertapenem Carbapenems (meropenem, imipenem)	5.5	4.5	4.6	4.7	5.1	3.8	4.0	3.0	3.9
Ertapenem	4.3	4.8	5.2	4.4	5.6	3.3	3.4	3.1	3.1
Ceftriaxone	35	34.7	32.2	30.8	30.9	28.4	28.9	24.1	24.4

**Table 3:** Results of the Interrupted Time Series Segmented Regression Analysis.

Antimicrobial Group	Baseline Trend (β_1_)	β_1_ *p*-Value	Level Change (β_2_)	β_2_ *p*-Value	Trend Change (β_3_)	β_3_ *p*-Value	R^2^
Total	−2.534	0.342	5.819	0.587	−2.730	0.462	0.714
Non-ertapenem Carbapenems (meropenem, imipenem)	−0.165	0.243	0.563	0.326	−0.131	0.496	0.695
Ertapenem	0.234	0.281	0.338	0.692	−0.714	**0.043**	0.623
Ceftriaxone	−1.516	**0.005**	0.781	0.615	−0.023	0.965	0.956

Note: β_1_ represents the baseline trend (DDD/100 bed-days per quarter); β_2_ represents the immediate level change at intervention; β_3_ represents the change in trend post-intervention. Bold *p*-values indicate statistical significance (*p* < 0.05).

**Table 4:** Number of patients identified with ASB and unnecessary antibiotic prescribed per month. Thirty-day return visit to the Emergency Department for Urinary Tract Infection with the same bacteria, thirty-day return visit to the Emergency Department for Urinary Tract Infection with any bacteria, and thirty-day mortality in both periods.

Outcomes	Pre-Intervention Group (n = 93)	Intervention Group (n = 102)	*p*-Value
Primary outcome:			
Number of patients identified with ASB and unnecessary antibiotic prescribed per month Median (IQR*)	19 (16–26)	9 (9–13)	0.018
Secondary outcomes (clinical safety):			
30-day UTI return visit to the ED (any bacteria)	11 (11.8%)	6 (5.9%)	0.225
30-day UTI return visit to the ED (same bacteria)	8 (8.6%)	2 (2%)	0.076
30-day mortality	8 (8.6%)	5 (4.9%)	0.455

IQR*: interquartile range.

**Table 5:** Summary of Antimicrobial Stewardship activities implemented in the Emergency Department.

Activity	Description
Education sessions	They were delivered by the AMS team, with active participation of Infectious Diseases Physicians, Clinical Pharmacists, and nursing staff. - ED prescribers: 23 May 2024; one-hour session at 08:00. - Nursing staff: 21–23 May 2024; 30 min sessions delivered at different times to cover all shifts (morning, afternoon, evening, and night).
Staff attendance	60% of ED prescribers (sessions). 90% of nursing staff. All material was shared later by email to 100% of ED prescribers and nursing staff.
Content covered	Definition of ASB and differentiation from UTI, IDSA 2019 recommendations and implementation in the ED, deprescribing algorithms, correct UC request criteria, appropriate UC sampling
Audit and feedback	July 2024–June 2025. Weekday proactive clinical assessment case-by-case was performed at 12:00: 15 min verbal feedback face-to-face discussion with ED prescribers and documentation of recommendations in the electronic medical record.

## Data Availability

The original contributions presented in this study are included in the article. Further inquiries can be directed to the corresponding authors.

## Abbreviations

ASB	Asymptomatic bacteriuria
UTI	Urinary tract infection
ED	Emergency department
DDD	Daily Define Dose
UC	Urine Culture
CRP	C Reactive Protein
AMS	Antimicrobial Stewardship
SD	Standard Deviation
IQR	Interquartile range
